# ZSM-5-Confined Fe-O_4_ Nanozymes Enable the Identification of Intrinsic Active Sites in POD-like Reactions

**DOI:** 10.3390/nano15141090

**Published:** 2025-07-14

**Authors:** Gaolei Xu, Yunfei Wu, Guanming Zhai, Huibin Ge

**Affiliations:** 1 Department of Human Anatomy, School of Basic Medicine, Zhengzhou University, Zhengzhou 450001, China; zimeng@zzu.edu.cn (G.X.); wuyf@stu.zzu.edu.cn (Y.W.);; 2 Interdisciplinary Research Center of Biology & Catalysis, School of Life Sciences, Northwestern Polytechnical University, Xi’an 710072, China

**Keywords:** isolated Fe-O_4_ sites Al-OH, medium acid sites, nanozymes, POD-like reaction

## Abstract

As widely used peroxidase-like nanozymes, Fe-based nanozymes still suffer from an unclear reaction mechanism, which limits their further application. In this work, through alkaline treatment and then the replacement or occupation of strong acid sites by isolated Fe species, porous ZSM-5-confined atomic Fe species nanozymes with separated medium acid sites (Al-OH) and isolated Fe-O_4_ sites were prepared. And the structure and the state of Fe-O_4_ confined by ZSM-5 were determined by AC-HAADF-STEM, XPS, and XAS. In the oxidation of 3, 3′, 5, 5′-tetramethylbenzidine (TMB) by the hydrogen peroxide (H_2_O_2_) process, the heterolysis of H_2_O_2_ to ∙OH mainly occurs at the isolated Fe-O_4_ sites, and then the generated ∙OH can spill over to the Al-OH sites to oxidize the adsorbed TMB. The synergistic effect between Fe-O_4_ sites and medium acid sites can significantly benefit the catalytic performance of Fe-based nanozymes.

## 1. Introduction

Since the first discovery that Fe_3_O_4_ nanoparticles (NPs) can exhibit intrinsic peroxidase (POD)-like activity to replace enzymes in nature, nanozymes, nanomaterial-based artificial enzymes, have attracted greatly increasing attention due to their high activity, low cost, convenience for mass production, and effectiveness in harsh conditions [1,2,3,4,5,6,7,8,9,10]. Although a variety of nanozymes have been synthesized, studied, and even applied, Fe-based nanozymes, especially Fe_3_O_4_ nanozymes, are also considered some of the most important POD-like artificial enzymes, since Fe is an essential element for humans [11,12,13]. The catalytic mechanisms of various Fe-based nanozymes have been studied and are thought to occur on the surface or at the interface of Fe-based nanozymes, but not at the leaching Fe ions in a reaction solution [14,15,16,17]. However, because of the limitations of characterization techniques and the complexity of nanozymes’ microstructure, clearly identifying active sites is still difficult, further hindering the accurate design of highly active nanozymes on an atomic scale to mimic enzymes in nature.

In the POD-like reaction process, the main function of nanozymes is to decompose H_2_O_2_ to ∙OH, which is the basis for determining whether a nanomaterial is a POD-like nanozyme [18,19]. And then, the generated ∙OH can be quantified through the colorimetric oxidation reaction of 3, 3′, 5, 5′-tetramethylbenzidine (TMB). Thus, most previous studies have mainly focused on the decomposition of H_2_O_2_ but ignored the TMB oxidation process, resulting in a failure to identify the real active sites and further our understanding of the catalytic mechanism. Fortunately, some scientists have begun to pay attention to the interaction between substrates and catalysts [20,21]. The properties of the supports can significantly affect the adsorption behavior of TMB, thereby affecting their activity. Taking the heme-like Fe1/AC (activated carbon) single-atom catalyst as an example [22], H_2_O_2_ is mainly decomposed at Fe-N_4_ sites, while the surface carboxyl group on the AC surface can act as a capturing group for the adsorption of the substrate, which determines the activity of the Fe1/AC catalyst. Our previous work also demonstrated the importance of support in Pt-TiO_2_-based catalysts [23], in which the surface of TiO_2_ represents the active site for the adsorption and oxidation of the substrate.

Thus, in order to avoid structural complexity and reveal the real active sites of Fe-based nanozymes, an atomically dispersed Fe nanozyme should be employed. Considering that MFI-type zeolites have a unique structure, they are beneficial for establishing the existence of single atoms [24,25]. Meanwhile, the properties of MFI-type zeolites can also be easily regulated through pretreatment. Thus, MFI-type zeolites represent a suitable support to prepare atomically dispersed Fe nanozymes. In this work, through alkaline treatment and then the replacement or occupation of strong acid sites by isolated Fe species, porous ZSM-5-confined isolated Fe nanozymes with separated medium sites (Al-OH) and atomic Fe sites were prepared. In a peroxidase-like reaction, the heterolysis of H_2_O_2_ to ∙OH occurs at the isolated Fe sites, and the generated ∙OH can spill over to the medium sites to oxidize TMB. The synergy between isolated Fe sites and acid sites is beneficial for the catalytic performance of Fe-based nanozymes.

## 2. Experimental Section

### 2.1. Nanozyme Preparation

Commercial ZSM-5 zeolites (ZSM-5-21, in which 21 is the ratio of SiO_2_/Al_2_O_3_) were first treated with 0.5 M NaOH solution under stirring at 50 °C for 30 min and then washed twice with 0.5 M NH_4_OH solution to replace the Na^+^. After filtering and drying at 80 °C, porous ZSM-5 was obtained. Here, porous ZSM-5 represents the formation of large through-holes in ZSM-5 after pretreatment with the NaOH solution. Second, iron nitrate with different concentrations was impregnated into the porous ZSM-5. After drying at 80 °C for 3 h, calcination at 400 °C for 12 h, and then reduction at 450 °C with a mixture of 10% H_2_/Ar (50 mL∙min^−1^) for 60 min, nanozymes were obtained and named Fe(*n*)-porousZSM-5, in which *n* represents the content of Fe from 0.1 wt% to 1 wt%.

### 2.2. Characterization of Nanozymes

Electron spin resonance (ESR) experiments were performed by using a Bruker ELEXSYS E500 CW ESR spectrometer (Bruker, Billerica, MA, USA) with a Bruker ER4122 super-high-Q cavity with a liquid nitrogen flow insert. N_2_ adsorption–desorption experiments were performed on the Micromeritics ASAP-2460 physical adsorption apparatus (Micromeritics, Atlanta, GA, USA). Prior to measurement, the nanozymes were degassed at 280 °C for 6 h. Fe K-edge absorption spectra were obtained using the BL14W1 beamline of the Shanghai Synchrotron Radiation Facility (SSRF), Shanghai Institute of Applied Physics, Chinese Academy of Sciences. This beamline adopted a fixed-exit double-crystal Si (111) monochromator with X-ray energy ranging from 5 to 23 keV. Fe foil, FeO, and Fe_2_O_3_ were used as the reference samples and measured in the transmission mode, and the nanozyme was measured in the fluorescence mode. The Demeter system was used to analyze the data of XAFS. In the Demeter system (https://bruceravel.github.io/demeter/, accessed on 1 July 2025), Athena software was used to calibrate the energy scale, correct the background signal, and normalize the intensity.

NH_3_-TPD experiments were conducted on the TP-5080-B chemical adsorption instrument of Tianjin Xianquan Instrument Co., Ltd., Tianjin, China. The nanozyme was treated by He (10 °C∙min^−1^) at 300 °C for 1 h to remove the impurity on the surface. After being cooled to 50 °C, the samples were exposed to a mixture of 7% NH_3_/He (30 mL∙min^−1^) for 60 min, followed by He (30 mL∙min^−1^) purging for 1 h, and then heated to 700 °C at a rate of 10 °C∙min^−1^.

### 2.3. Catalytic Evaluation

Experiments were carried out in a 2 mL quartz cuvette with vibration at 37 °C. First, 20 μL TMB in DMSO solution (25 mmol∙L^−1^) and 20 μL H_2_O_2_ at 25 mmol∙L^−1^ were added into the quartz cuvette. Then, 940 μL NaAc-HAC buffer (0.2 mmol∙L^−1^) was added to complement the reaction system to 1 mL and keep the solution at a consistent pH of 3.6. The nanozyme was added to the reaction system at a concentration of 20 μg∙mL^−1^. In the experiment, the signal at 652 nm was recorded every 40 s by a Nanodroup (Thermo Fisher Scientific, Waltham, MA, USA).

The concentrations of the product were calculated according to the following formula.

*C*_oxTMB_ = A∙*ε*^−1^∙L^−1^

where A represents the absorbance value detected by a spectrophotometer at 652 nm. *ε* is the molar absorption coefficient of oxTMB, which is 39,000 L∙mol^−1^∙cm^−1^. L is the optical path. *C*_oxTMB_ represents the concentration of oxTMB. According to the above formula, the concentrations of oxTMB in the reaction system were obtained.

The TOF was calculated based on the content of Fe and the amount of converted TMB from the 50th second to the 150th second, during which time the contribution of H_2_O_2_ was sufficient. Unless otherwise specified, the calculation was carried out according to the above method.

## 3. Results and Discussion

### 3.1. The Structure of Nanozymes

**Figure 1:**
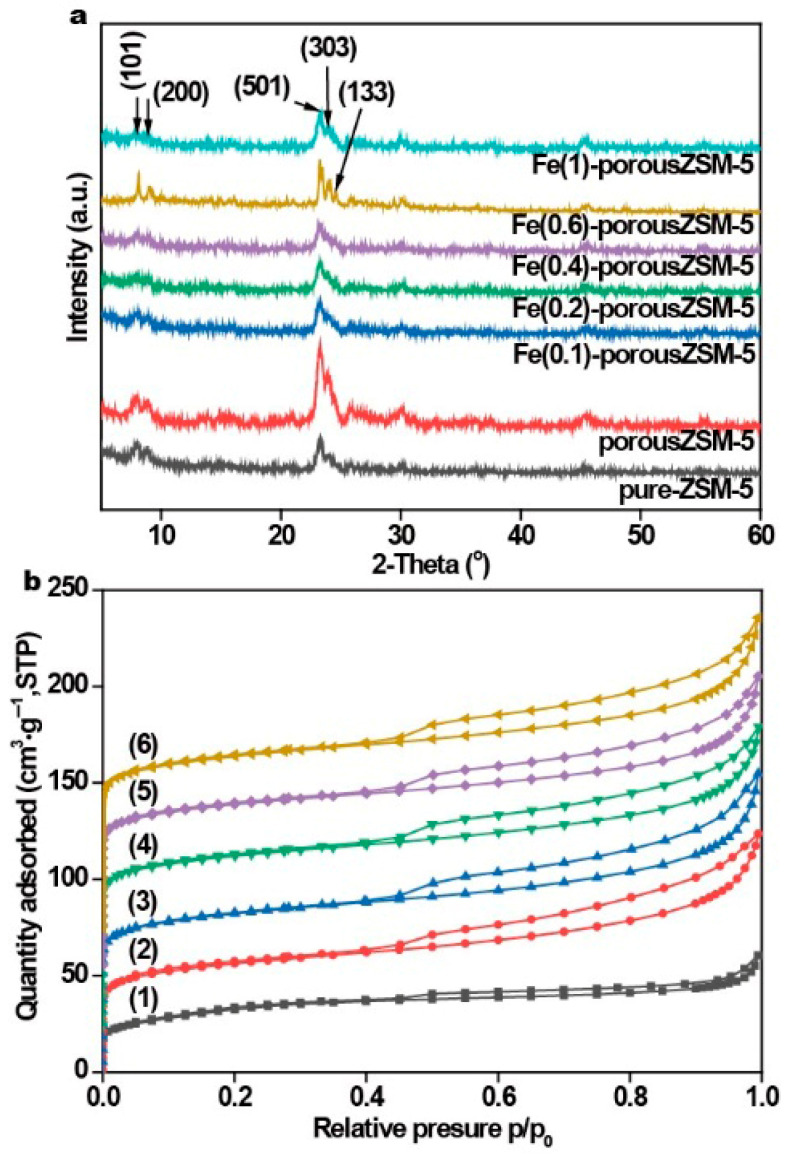
(**a**) XRD patterns and (**b**) nitrogen adsorption–desorption isotherms of the relative nanozymes: (1) pure ZSM-5, (2) porousZSM-5, (3) Fe(0.1)-porousZSM-5, (4) Fe(0.2)-porousZSM-5, (5) Fe(0.6)-porousZSM-5, and (6) Fe(1)-porousZSM-5.

The powder X-ray diffraction measurements (XRD, Figure 1a) show that two typical peaks in 2θ = 7°–9° and three distinctive peaks in 2θ = 23°–25° are attributed to the (101), (200), (501), (303), and (133) planes of ZSM-5 [26,27]. The results reveal that each sample exhibits the typical diffraction peaks of the MFI-type framework structure, indicating that the ZSM-5 zeolite framework is not destroyed after alkaline treatment. However, no characteristic peaks of Fe species are observed, indicating a low content or high dispersion of Fe species in all the Fe(*n*)-porousZSM-5 samples.

The porosity of the Fe(*n*)-porousZSM-5 samples was determined by nitrogen adsorption–desorption measurements. Figure 1b shows that all the samples present the type IV isotherm. The high N_2_ uptake at low *P*/*P*_0_ is related to the micropore structure. And the improved N_2_ uptake at high *P*/*P*_0_ is related to the capillary condensation of nitrogen within the huge space. The H2-type hysteresis loop with an abrupt step around *P*/*P*_0_ = 0.45 in the desorption branch observed on all the zeolites indicates that most of the zeolite structures were not destroyed. The BET surface area and the pore volume of the zeolites are shown in Table S1 and Figure S1. Compared with pure ZSM-5, all surface area, pore volume, and pore width values increased significantly for the Fe(*n*)-porousZSM-5 samples (*n* = 0, 0.1, 0.2, 0.6, and 1). And a new pore with about 2.4 nm appeared in all the zeolites after the alkaline treatment. The results reveal that gradient pores were formed after the alkaline treatment. Meanwhile, with the increase in the Fe content, the surface area and the pore volume were almost the same, suggesting that the Fe species are highly dispersed in the porous zeolites. This conclusion can also be supported by the UV–Vis spectra (Figure S2), in which no Fe nanoparticles are observed.

**Figure 2:**
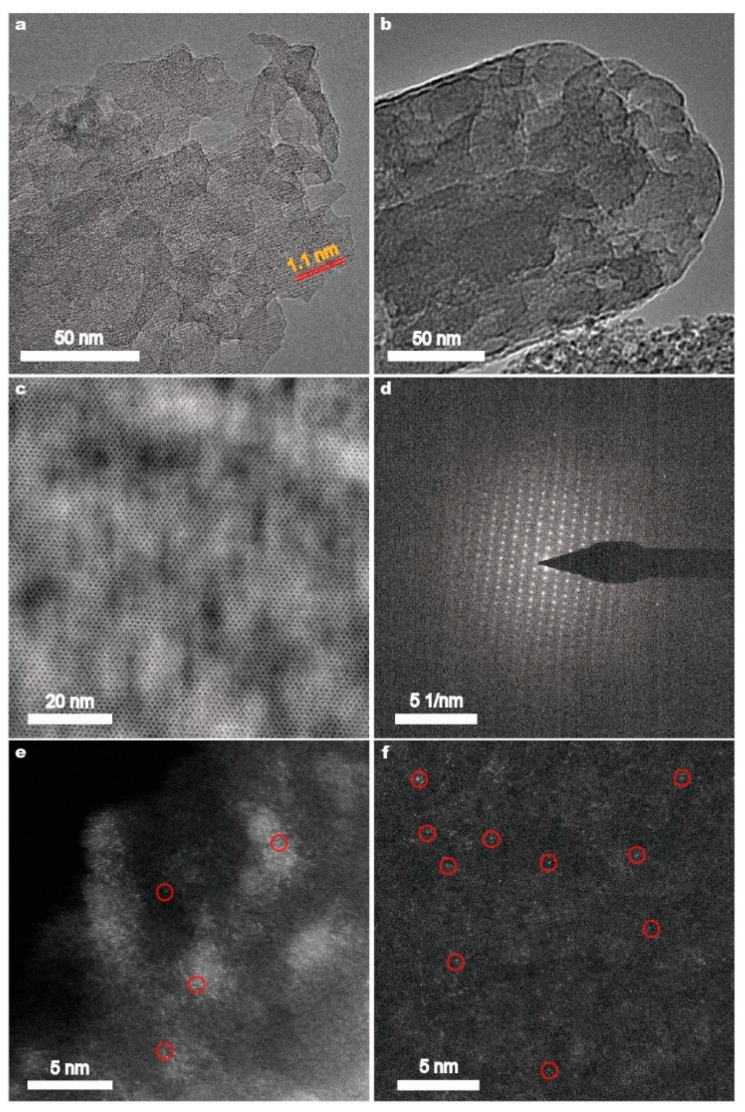
TEM images of the Fe(0.2)-porousZSM-5 (**a**) and Fe(0.6)-porousZSM-5 (**b**) nanozymes. The MFI structure (**c**) and SAED pattern (**d**) of the Fe(0.6)-porousZSM-5 nanozyme. AC-HAADF-STEM images of the Fe(0.2)-porousZSM-5 (**e**) and Fe(0.6)-porousZSM-5 (**f**) nanozymes. The bright spots in the red circles are Fe atoms.

TEM and HRTEM were employed to investigate the structure of the Fe(*n*)-porousZSM-5 samples. Numerous pores are observed in the Fe(*n*)-porousZSM-5 samples (Figure 2a,b and Figure S3). And a 1.1 nm lattice distance, which is assigned to the (101) planes of ZSM-5, is also observed in Figure 2a. In Figure 2c,d, the topological structure and the SAED pattern of the MFI zeolites are found. The results reveal that the gradient pores formed with the ZSM-5 zeolite framework were undestroyed after the alkaline treatment. No obvious nanoparticles or clusters could be observed for the Fe(0.2)-porousZSM-5, Fe(0.6)-porousZSM-5, and Fe(1)-porousZSM-5 samples from their TEM images in Figure 2a–c and Figure S3, revealing the high dispersion of Fe species in all the Fe(*n*)-porousZSM-5 samples. With increasing magnification, a large number of isolated and recognizable bright spots are highly dispersed in both the Fe(0.2)-porousZSM-5 and Fe(0.6)-porousZSM-5 nanozymes, revealing that Fe exists as a single atom in the two nanozymes (Figure 2e,f). Several clusters are observed in the Fe(1)-porousZSM-5 samples (Figure S3). To clarify whether they belong to Fe species, element energy dispersive spectrometer (EDS) experiments were carried out. Obviously, the bright area in Figure S3c does not match with the distribution of the Fe element (Figure S3d). Thus, Fe is also highly dispersed in Fe(1)-porousZSM-5.

**Figure 3:**
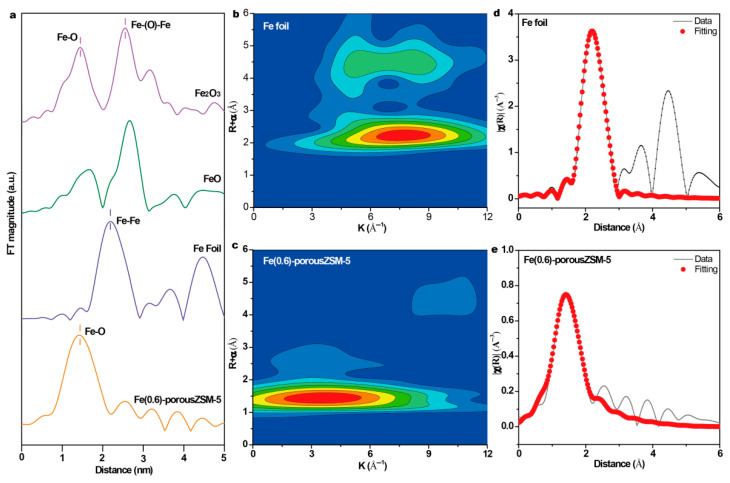
(**a**) The Fe *K*-edge Fourier-transformed *k*^2^-weighted EXAFS spectra in R space. (**b**,**c**) The wavelet transforms from EXAFS of Fe foil and Fe(0.6)-porousZSM-5, respectively. (**d**,**e**) The corresponding fitting R-space curves of Fe foil and Fe(0.6)-porousZSM-5, respectively.

**Figure 4:**
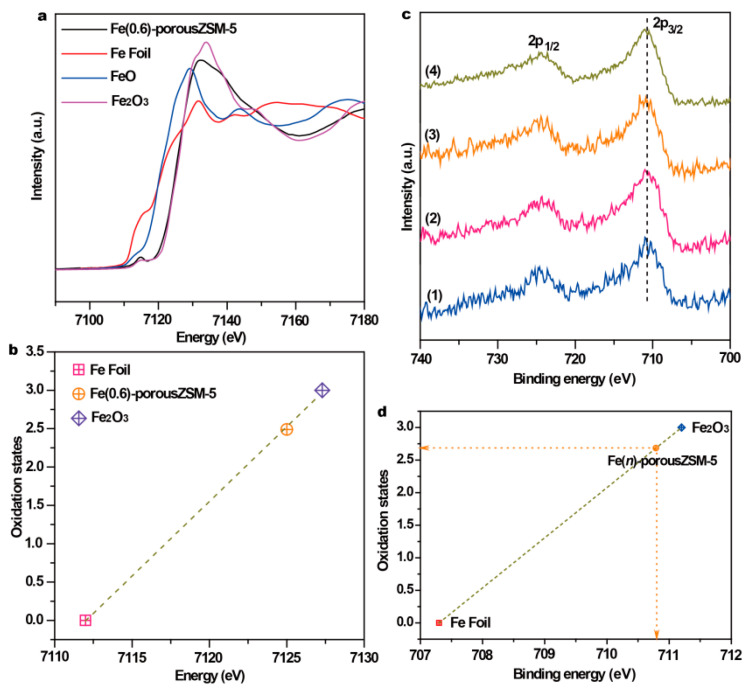
(**a**) The Fe *K*-edge XANES spectra and (**b**) the corresponding oxidation state of Fe in Fe(0.6)-porousZSM-5. (**c**) XPS spectra of the nanozymes and (**d**) the corresponding oxidation state of Fe in Fe(*n*)-porousZSM-5. (1) Fe(0.2)-porousZSM-5, (2) Fe(0.4)-porousZSM-5, (3) Fe(0.6)-porousZSM-5, and (4) Fe(1)-porousZSM-5.

To obtain the coordinating structure information of Fe species in the porous zeolites, extended X-ray absorption fine structure spectra (EXAFS) of the Fe(0.6)-porousZSM-5 sample and the reference samples were measured. The spectra in Figure 3a show that there is only the first shell Fe-O at 1.46 Å (without phase shift) in Fe(0.6)-porousZSM-5, verifying that only isolated Fe species exist. Meanwhile, the wavelet transforms from EXAFS (Figure 3b,c) show that there is a large difference between the Fe foil and Fe(0.6)-porousZSM-5, further reflecting the dispersion of Fe single atoms. In addition, the corresponding EXAFS fitting curves (Figure 3e and Table S2) indicate two separated Fe-O shells, a distance of 1.88 Å, with a coordination number of 2.17, and a distance of 2.07 Å, with a coordination number of 2.33, corresponding to Fe-O-Al/Si and Fe-OH/H_2_O, respectively [28,29]. The results imply that the single Fe atom is only coordinated with about four oxygen atoms (Fe-O_4_). The electronic structure of Fe in the Fe(0.6)-porousZSM-5 sample was also identified by normalized X-ray absorption near-edge structure (XANES) spectroscopy. As shown in Figure 4a, compared with the Fe foil, Fe(0.6)-porousZSM-5 shows a higher energy, close to Fe_2_O_3_. And an obvious pre-edge peak at 7114.8 eV, generated from both the allowed electric quadrupole and 3d-4p mixing electric dipole over 1s to 3d transition [22,30], is exhibited in Fe(0.6)-porousZSM-5. Then, the Fe state was quantified. As shown in Figure 4b, the average oxidation state of Fe in Fe(0.6)-porousZSM-5 is about +2.5, which is similar to that in Fe_3_O_4_. The state of Fe was further provided by XPS spectra. As shown in Figure 4c, all the Fe(*n*)-porousZSM-5 samples show a doublet with a 2p_3/2_ binding energy of 710.7 eV. In Figure 4d, taking the Fe foil and Fe_2_O_3_ as the references, the average oxidation state of Fe in Fe(*n*)-porousZSM-5 is about +2.6, which is consistent with the result from EXAFS.

**Figure 5:**
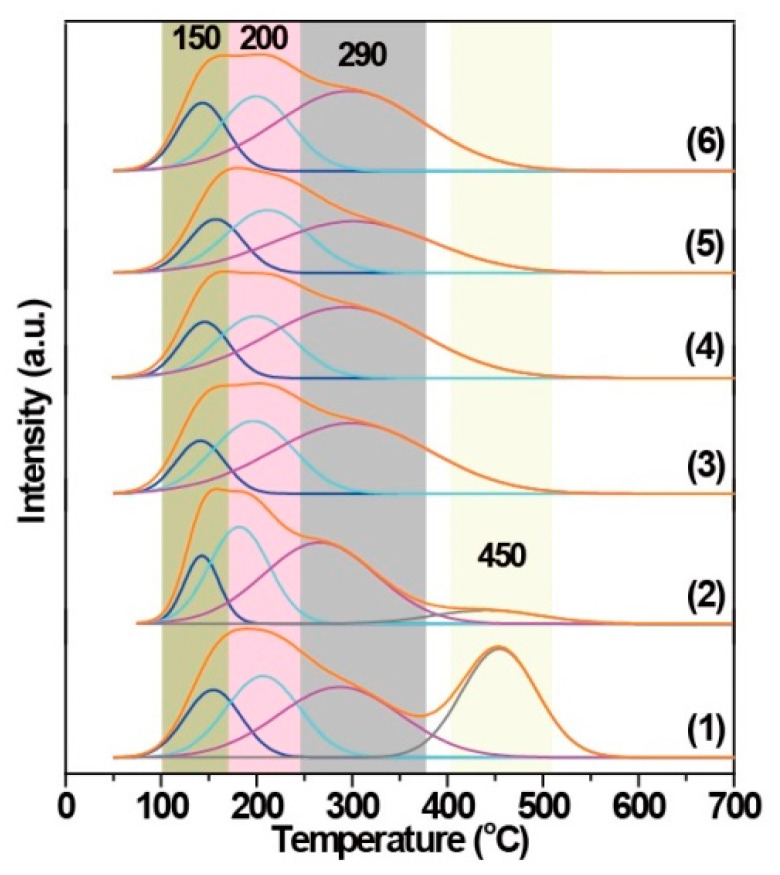
NH_3_-TPD spectra of the nanozymes: (1) porousZSM-5, (2) Fe(0.1)-porousZSM-5, (3) Fe(0.2)-porousZSM-5, (4) Fe(0.4)-porousZSM-5, (5) Fe(0.6)-porousZSM-5, and (6) Fe(1)-porousZSM-5.

Figure 5 presents the NH_3_-TPD curves of the related Fe(*n*)-porousZSM-5 samples. The porous ZSM-5 sample shows two broad NH_3_ desorption peaks, a low-temperature peak resulting from NH_3_ desorption of weak/medium acid sites [31,32], and a high-temperature peak contributing to NH_3_ desorption from strong acid sites (bridging Si-O(H)-Al in the framework) [33]. Obviously, the peak at high temperature decreases significantly in the Fe(0.1)-porousZSM-5 nanozyme. And with the introduction of more iron, the peak at 450 °C even vanished, indicating that the strong acid sites are replaced or occupied by Fe to form Fe-O-Al/Si [34,35], which is consistent with the EXAFS fitting results. To better identify the acid sites, the low-temperature peak can be deconvoluted into three peaks for all the Fe(*n*)-porousZSM-5 samples [36]. The first one, at around 150 °C, is related to physically adsorbed ammonia [37,38]. The second peak, at around 200 °C, can be attributed to the ammonia weakly adsorbed on independent Al_2_O_3_ (weak acid sites) [39]. The last peak, at around 290 °C, belongs to NH_3_ adsorbed on extra-framework aluminum species (Al-OH) in the zeolite (medium acid sites) [39]. Estimated from the area of the three deconvoluted desorption peaks, the acidity of each peak was calculated and is presented in Table 1. Both the total acidity and the medium acid sites decrease progressively with the increase in the Fe content from 0.1 wt% to 0.6 wt%, suggesting that part of the medium acid sites are occupied by iron with no/slight new acid sites formation. When more Fe is loaded, the medium acid sites increase inversely, suggesting the additional Fe presents as FeOx clusters [36,37,38].

### 3.2. Nanozymes Activity

**Figure 6:**
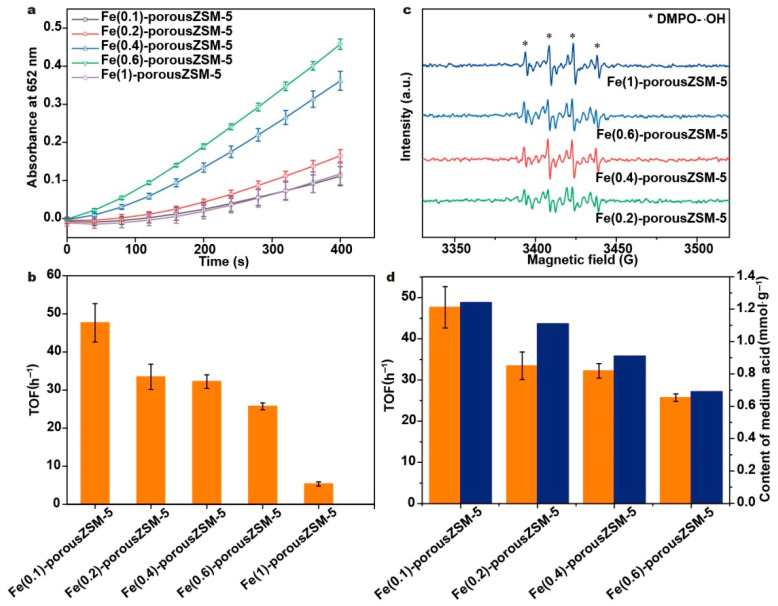
(**a**) Time on stream curves of the TMB oxidation reaction catalyzed by the Fe(*n*)-porousZSM-5 nanozymes. The experiments were tested with a temperature of 37 °C, a pH of 3.6, a H_2_O_2_ concentration of 0.5 mmol L^−1^, a TMB concentration of 0.5 mmol L^−1^, and the nanozyme at 20 μg. (**b**) The TOF of the Fe(*n*)-porousZSM-5 nanozymes. (**c**) ESR characterization of different nanozymes. (**d**) The relationship between the TOF (orange) and the amount of medium acid sites (blue).

The peroxidase-like activity of the Fe(*n*)-porousZSM-5 nanozymes was evaluated with the oxidation of TMB by hydrogen peroxide. The reaction process was carried out at 37 °C with a pH of 3.6, a H_2_O_2_ concentration of 25 mmol∙L^−1^, a TMB concentration of 0.5 mmol∙L^−1^, and the quality of nanozymes at 20 μg∙mL^−1^. As shown in Figure 6a, the absorbance intensity increases first and then drops with the increasing Fe content. And the Fe(0.6)-porousZSM-5 nanozyme exhibits the best activity. Meanwhile, the steady-state kinetics of Fe(0.6)-porousZSM-5 towards TMB and H_2_O_2_ was conducted (as shown in Figure S4). The calculated values of *K_m_* and *V_max_*, based on the Michaelis–Menten equation, are 12.6 mM and 1.3 × 10^−6^ mMs^−1^ for TMB and 13.5 mM and 3.3 × 10^−7^ mMs^−1^ for H_2_O_2_. To reveal the intrinsic activity, the normalized turnover frequency (TOF) based on the total Fe content is used to describe the peroxidase-like activity of the nanozymes (Figure 6b). As expected, the Fe(0.1)-porousZSM-5 nanozyme shows the best TOF of 48 h^−1^. And with the increasing Fe content, the TOF decreases progressively.

### 3.3. Discussion

A pure porous ZSM-5 sample was used for the catalytic oxidation of TMB by H_2_O_2_. A negligible activity is observed (Figure S5). Normally, the unabsorbed molecules of TMB can be oxidized by the hydroxyl radical in the current reaction conditions. Thus, one can conclude that it is Fe but not ZSM-5 that is the active site for the decomposition of H_2_O_2_. An ESR experiment was performed, as shown in Figure 6c and Figure S6. The typical ESR signals assigned to the hydroxyl radical (∙OH) are observed in all the Fe(*n*)-porousZSM-5 nanozymes [40,41]. Obviously, there is almost no hydroxyl radical in the porous ZSM-5 sample. And the intensity of the hydroxyl radical increases with the increasing Fe content, further proving that H_2_O_2_ is decomposed into ∙OH at the Fe-O_4_ sites. It should be noted that the intensity of the hydroxyl radical from Fe(1)-porousZSM-5 is slightly higher than that from Fe(0.6)-porousZSM-5, but the activity of Fe(1)-porousZSM-5 is much lower than that of Fe(0.6)-porousZSM-5 (Figure 6a). Meanwhile, both Fe(0.6)-porousZSM-5 and Fe(0.4)-porousZSM-5 have a similar ability to generate hydroxyl radical (Figure 6c), but the activity of Fe(0.6)-porousZSM-5 is much higher than that of Fe(0.4)-porousZSM-5 (Figure 6a). The results indicate that the TMB oxidation step, rather than the OH radical generation, is the rate-determining step.

Assuming that isolated Fe-O_4_ sites are the only active sites, the Fe(0.1)-porousZSM-5 and Fe(0.6)-porousZSM-5 nanozymes should present a similar TOF because of the similar Fe in them. However, compared to Fe(0.1)-porousZSM-5, the TOF of Fe(0.6)-porousZSM-5 decreases by about 30%, revealing that Fe-O_4_ sites are not the active sites for the oxidation of TMB. When correlating the activity and the content of acid sites (Figure 6d and Figure S7), it is easy to observe that the activity is close to a linear relationship with the amount of medium acid sites, indicating that the TMB oxidation process mainly occurs at the medium acid sites (Al-OH). Meanwhile, the Fe(1)-porousZSM-5 nanozyme has a large number of medium acid sites but presents the lowest TOF of all the nanozymes. Because of the effective decomposition of hydrogen peroxide to ∙OH for the Fe(1)-porousZSM-5 nanozyme (Figure 6c), one can conclude that the extra-framework aluminum sites (Al-OH) in the medium acid sites are the active sites for the oxidation of TMB. Thus, in the Fe(n)-porousZSM-5 nanozymes, H_2_O_2_ is first decomposed to ∙OH at the isolated Fe-O_4_ sites. Then, the generated ∙OH can migrate to the extra-framework aluminum sites (Al-OH) to oxidize the adsorbed TMB. The synergy between the isolated Fe and the medium acid sites can significantly improve the peroxidase-like activity.

## 4. Conclusions

In summary, separating the different active sites in metal–zeolite nanozymes provides an opportunity to reveal the intrinsic active sites. In this work, through alkaline treatment, porous ZSM-5 with medium acid sites belonging to Al-OH was prepared. Then, after the replacement or occupation of the strong acid sites by isolated Fe species, the nanozymes with isolated Fe-O_4_ sites and certain amounts of medium acid sites were obtained. In the POD-like reaction, the heterolysis of H_2_O_2_ to ∙OH occurs at the Fe-O_4_ sites, and the generated ∙OH can spill over to the Al-OH sites to oxidize TMB. The synergy between the isolated Fe-O_4_ sites and Al-OH sites can significantly improve the peroxidase-like activity. This work has provided an efficient method to separate and identify different active sites, which is beneficial for the understanding of the intrinsic synergistic mechanism between each active site.

## Figures and Tables

**Table 1:** The relative data for the Fe(*n*)-porousZSM-5 nanozymes.

Nanozyme	Content ^a^ (wt%)	Acidity by Strength ^b^ (mmol∙g^−1^)		
		Strong Acidity	Medium Acidity	Weak and Physical Acidity
porousZSM-5		0.92	0.97	1.10
Fe(0.1)-porousZSM-5	0.11	0.15	1.24	0.91
Fe(0.2)-porousZSM-5	0.21	0	1.11	0.85
Fe(0.4)-porousZSM-5	0.40	0	0.91	0.57
Fe(0.6)-porousZSM-5	0.62	0	0.69	0.69
Fe(1)-porousZSM-5	0.97	0	0.75	1.01

^a^ The content of iron was obtained from icp-oes. ^b^ The density of acid sites was determined by NH_3_-TPD.

## Data Availability

The data are included within this article and the Supplementary Materials.

## Supplementary Materials

The following supporting information can be downloaded at https://www.mdpi.com/article/10.3390/nano15141090/s1: Scheme S1: The reaction process for the oxidation of TMB to oxTMB by H_2_O_2_; Figure S1: Micropore size distribution of the relative nanozymes; Figure S2: UV–vis spectra of the Fe-porousZSM-5 zeolites; Figure S3: TEM (a) and AC-HAADF-STEM (b) images of the Fe(1)-porousZSM-5 nanozyme (c) and (d) EDX mapping of Fe; Figure S4: The steady-state kinetic curves of the Fe(0.6)-porousZSM-5 nanozyme towards TMB (a) and H_2_O_2_ (b); Figure S5: The comparation of the activity between Fe(1)-porousZSM-5 and pure porousZSM-5; Figure S6: ESR curves of the Fe(0.2)-porousZSM-5 and porous ZSM-5 samples; Figure S7: The relationship between the TOF and the content of acid sites; Table S1: Textural properties of the related zeolites; Table S2: EXAFS data fitting results of the Fe *K*-edge of different samples. Refs [42,43,44] are cited in the supplementary materials.
