# Supplementary material for: ZSM-5-Confined Fe-O4 Nanozymes Enable the Identification of Intrinsic Active Sites in POD-like Reactions
Source: Nanomaterials (Basel). 2025 Jul 14;15(14):1090. doi: 10.3390/nano15141090 (PMC12298369; doi:10.3390/nano15141090)
Supplement: Supplementary file 1 [file nanomaterials-15-01090-s001.zip › nanomaterials-3687973-supplementary.pdf]

**Supporting information for**

# ZSM-5 confined Fe-$O_4$ nanozymes enable the identification of intrinsic active sites in POD-like reaction

Gaolei Xu[1], Yunfei Wu[1], Guanming Zhai[1], and Huibin Ge[2]*,

[1] Department of Human Anatomy, School of Basic Medicine, Zhengzhou University, zhengzhou, 450001, China. zimeng@zzu.edu.cn (G.X.); wuyf@stu.zzu.edu.cn (Y.W.); zgm202351010603@stu.zzu.edu.cn (G.Z.)

[2] Interdisciplinary Research Center of Biology & Catalysis, School of Life Sciences, Northwestern Polytechnical University, Xi'an 710072, China.

*Correspondence:gehuibin@nwpu.edu.cn

## Characterization of the nanozymes

Powder X-ray diffraction (XRD) spectra were obtained using Cu-Kα radiation. Continuous scans were collected over the 2θ range 5-90 ° with a step size of 0.5 ° for a dwell time of 3 s per step. The microstructure of the nanozyme was observed by Aberration-corrected high-angle annular dark-field scanning transmission electron microscopy (AC-HAADF-STEM, Themis, FEI) at an accelerating voltage of 200 kV. The X-ray photoelectron spectra (XPS) were collected using an ESCALab 250 X-ray photoelectron spectrometer with Al-Kα source. And the binding energy scale was referenced to the C 1s level of the carbon at 284.8 eV.

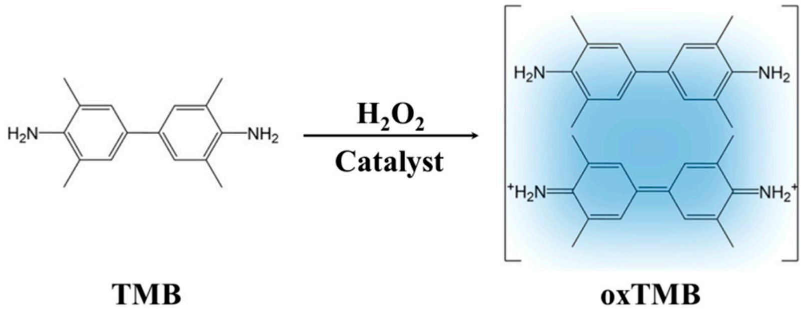


**Scheme S1** The reaction process for the oxidation of TMB to oxTMB by $H_2O_2$.

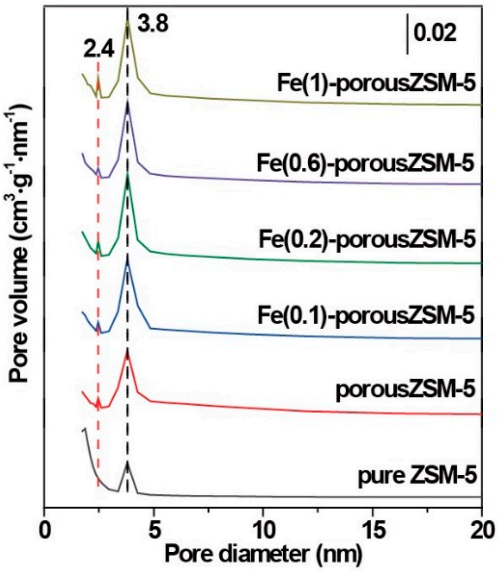


**Figure S1** Micropore size distribution of the relative nanozymes.

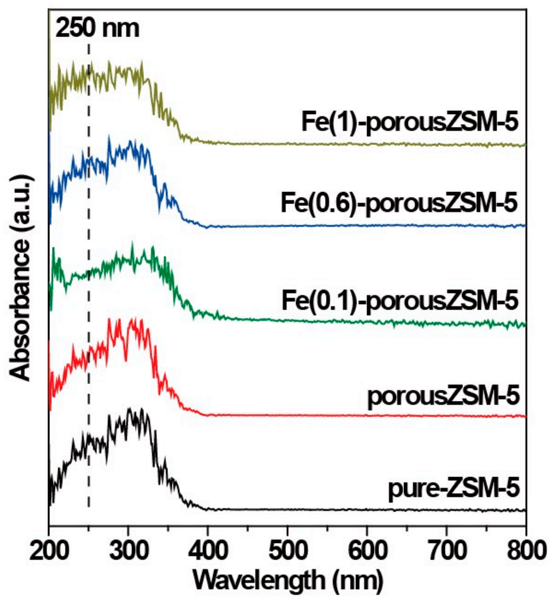


**Figure S2** UV–vis spectra of the Fe-porousZSM-5 zeolites.

N Normally, the bands around 250 nm are assigned to isolated $Fe^{3+}$ ions in tetrahedral or octahedral coordination in the Fe-ZSM-5 zeolites.[42, 43] And the peaks between 400-600 nm are attributed to $Fe_2O_3$ nanoparticles at the external surface of the zeolite crystal.[42, 44] Obviously, compared with pure ZSM-5 and porousZSM-5, board bands around 250 nm were observed after the introduction of Fe into the porousZSM-5. And no peaks between 400-600 nm were found in them. Thus the UV-vis spectra proved that only isolated Fe species existed in the Fe(n)-porousZSM-5 nanozymes. Meanwhile, the EXAFS has proved that an Fe atom is coordinated with about four oxygen atoms, thus the isolated Fe is in tetrahedral coordination.

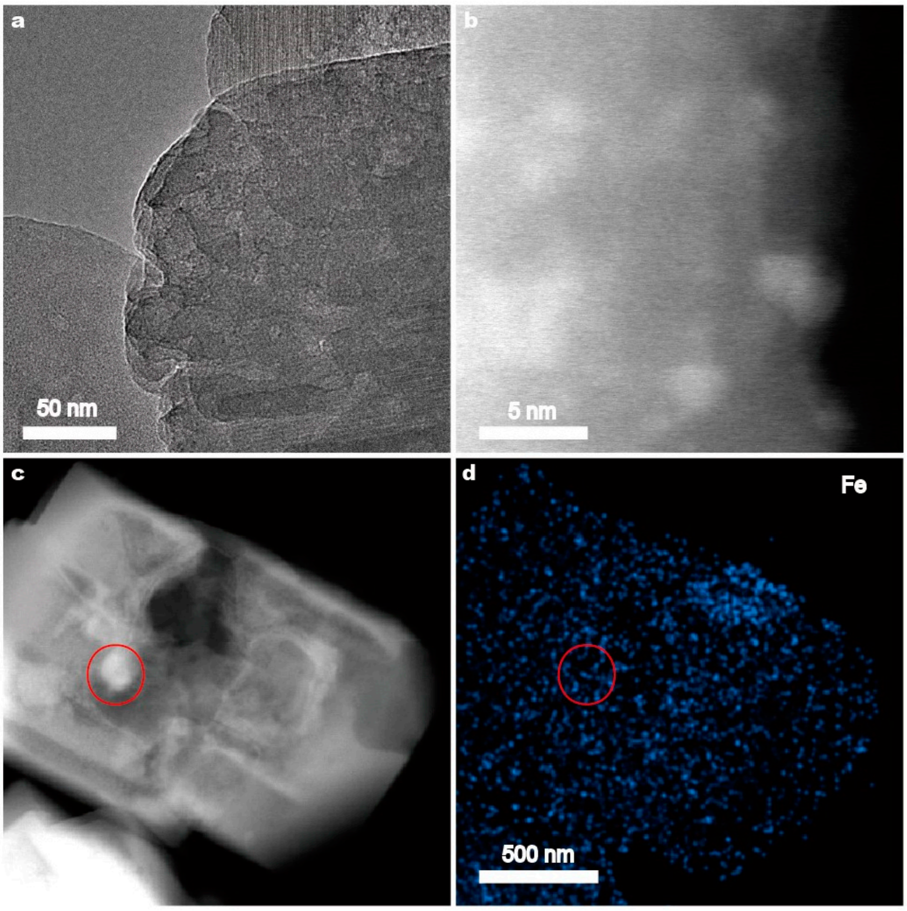


**Figure S3** TEM (a) and AC-HAADF-STEM (b) images of Fe(1)-porousZSM-5 nanozyme. (c) and (d) EDX mapping of Fe.

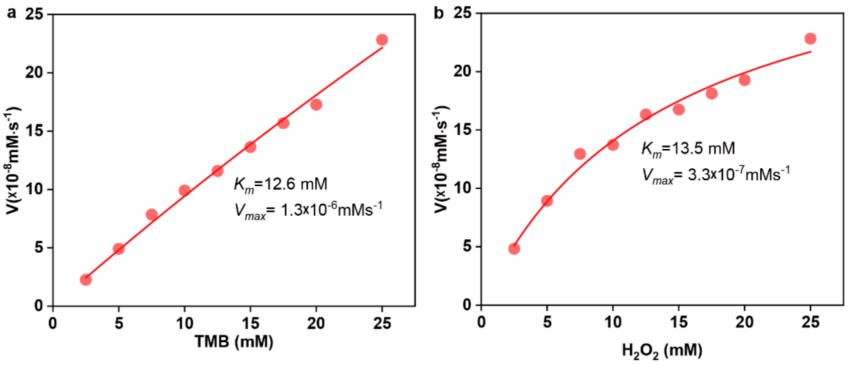


**Figure S4** The steady-state kinetic curves of Fe(0.6)-porousZSM-5 nanozyme towards TMB (a) and $H_2O_2$ (b).

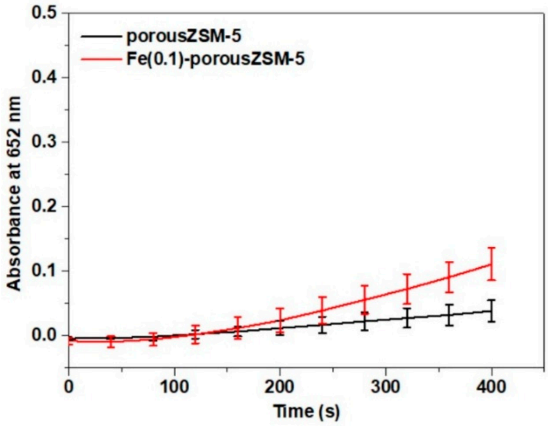


**Figure S5** The comparation of the activity between Fe(1)-porousZSM-5 and pure porousZSM-5.

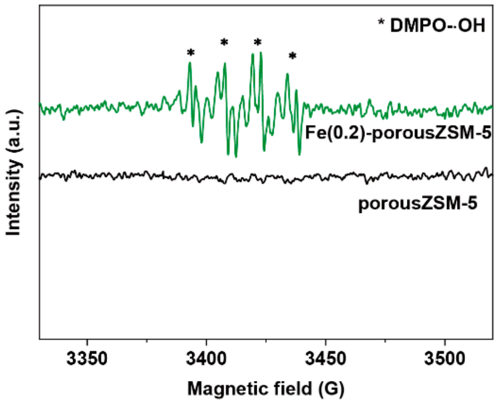


**Figure S6** ESR curves of Fe(0.2)-porousZSM-5 and porous ZSM-5 samples.

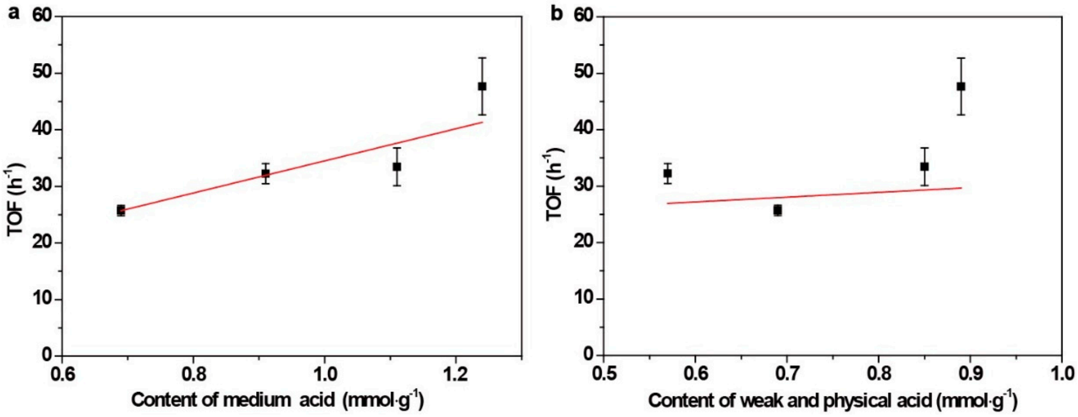


**Figure S7** The relationship between the TOF and the content of acid sites.

| **Table S1.** Textural properties of the related zeolites. | | | | | |
|---|---|---|---|---|---|
| Samples | Fe content (wt.%) | $S_{BET}$ ($m^2g^{-1}$) | $V_{micro}$ ($cm^3g^{-1}$) | $V_{meso}$ ($cm^3g^{-1}$) | Pore width (nm) |
| pure ZSM-5 | 0 | 252.1 | 0.08 | 0.09 | 2.59 |
| porousZSM-5 | 0 | 282.1 | 0.09 | 0.14 | 3.40 |
| Fe(0.1)-porousZSM-5 | 0.1 | 310.5 | 0.11 | 0.15 | 3.38 |
| Fe(0.2)-porousZSM-5 | 0.2 | 354.4 | 0.12 | 0.13 | 2.78 |
| Fe(0.6)-porousZSM-5 | 0.6 | 339.3 | 0.11 | 0.13 | 2.83 |
| Fe(1)-porousZSM-5 | 1.0 | 360.1 | 0.12 | 0.14 | 2.86 |

$S_{BET}$, BET surface area.

$V_{micro}$, micropore volume determined by t-plot.

$V_{meso}$, mesopore volume determined by $V_{total}$ -$V_{micro}$.

| **Table S2**. EXAFS data fitting results of the Fe K-edge of different samples. | | | | | |
|---|---|---|---|---|---|
| Sample | Shell | CN | *R* (Å) | $\sigma^2$×$10^3$(Å$^2$) | $\Delta E_0$ (eV) |
| Fe Foil | Fe-Fe1 | 8 | 2.46 | 4.96 | 5.07 |
| | Fe-Fe2 | 6 | 2.85 | 4.96 | 5.07 |
| Fe(0.6)-porousZSM-5 | Fe-O1 | 2.17 | 1.88 | 2.0 | -3.85 |
| | Fe-O2 | 2.33 | 2.07 | 2.0 | -0.04 |

*CN*, the coordination number for the absorber-backscatter pair; *R*, the average absorber-backscatter distance; $\sigma^2$, the Debye-Waller factor; $\Delta E_0$, the inner potential correction.

The data ranges used for data fitting in *K*-space ($\Delta K$) and *R*-space ($\Delta R$) are 3.0-9.2 Å$^{-1}$ and 1-2.9 Å, respectively.
